# *Anaplasma phagocytophilum* Activates NF-κB Signaling via Redundant Pathways

**DOI:** 10.3389/fpubh.2020.558283

**Published:** 2020-10-30

**Authors:** J. Stephen Dumler, Marguerite Lichay, Wan-Hsin Chen, Kristen E. Rennoll-Bankert, Jin-ho Park

**Affiliations:** ^1^Department of Pathology, F. Edward Hébert School of Medicine, Uniformed Services University for the Health Sciences, Bethesda, MD, United States; ^2^Division of Medical Microbiology, Department of Pathology, The Johns Hopkins University School of Medicine, Baltimore, MD, United States

**Keywords:** *Anaplasma phagocytophilum*, NF-κB, signaling, neutrophil, inflammation

## Abstract

*Anaplasma phagocytophilum* subverts neutrophil function permitting intracellular survival, propagation and transmission. Sustained pro-inflammatory response, recruitment of new host cells for population expansion, and delayed apoptosis are associated with prolonged nuclear presence of NF-κB. We investigated NF-κB signaling and transcriptional activity with *A. phagocytophilum* infection using inhibitors of NF-κB signaling pathways, and through silencing of signaling pathway genes. How inhibitors or silencing affected *A. phagocytophilum* growth, inflammatory response (transcription of the κB-enhanced genes *CXCL8* and *MMP9*), and NF-κB signaling pathway gene expression were tested. Among *A. phagocytophilum*-infected HL-60 cells, nuclear NF-κB p50, p65, and p52 were detected by immunoblots or iTRAQ proteomics. *A. phagocytophilum* growth was affected most by the IKKαβ inhibitor wedelolactone (reductions of 96 to 99%) as compared with SC-514 that selectively inhibits IKKβ, illustrating a role for the non-canonical pathway. Wedelolactone inhibited transcription of both *CXCL8* (*p* = 0.001) and *MMP9* (*p* = 0.002) in infected cells. Compared to uninfected THP-1 cells, *A. phagocytophilum* infection led to >2-fold down regulation of 64 of 92 NF-κB signaling pathway genes, and >2-fold increased expression in only 4. Wedelolactone and SC-514 reversed downregulation in all 64 and 45, respectively, of the genes down-regulated by infection, but decreased expression in 1 gene with SC-514 only. Silencing of 20 NF-κB signal pathway genes increased bacterial growth in 12 (*IRAK1, MAP3K1, NFKB1B, MAP3K7, TICAM2, TLR3, TRADD, TRAF3, CHUK, IRAK2, LTBR*, and *MALT1*). Most findings support canonical pathway activation; however, the presence of NFKB2 in infected cell nuclei, selective non-canonical pathway inhibitors that dampen *CXCL8* and *MMP9* transcription with infection, upregulation of non-canonical pathway target genes *CCL13* and *CCL19*, enhanced bacterial growth with *TRAF3* and *LTBR* silencing provide evidence for non-canonical pathway signaling. Whether this impacts distinct inflammatory processes that underlie disease, and whether and how *A. phagocytophilum* subverts NF-κB signaling via these pathways, need to be investigated.

## Introduction

*Anaplasma phagocytophilum* is an *Ixodes* spp. tick-transmitted zoonotic rickettsia of small mammals, cervids, and ruminants (1). Infection of co-evolved mammalian reservoirs often results in mild, subclinical, and persistent infections (2). However, granulocytic anaplasmosis is a mild to severe disease of humans, horses, and dogs, characterized in humans by fever, headache, myalgias, thrombocytopenia, leukopenia, and liver injury (1). This obligate intracellular rickettsial bacterium infects and propagates within granulocytes, largely neutrophils, in blood and bone marrow after it is acquired by tick bite and disseminated into the blood.

*A. phagocytophilum* evolved to survive within the antimicrobial confines of neutrophils by co-opting a number of pathways that regulate endosomal entry, vacuolar trafficking, and signals that lead to delayed apoptosis and reduced antimicrobial activities, while promoting an inflammatory environment that recruits new host cells to expand bacterial populations and inducing inflammatory cell injury as a mechanism for disease pathogenesis (3–5). The mechanisms by which *A. phagocytophilum* subverts its mammalian host cell, the neutrophil, to achieve all of these functions has been greatly studied, with a deep understanding of some microbial mechanisms and control over host functions. A key for understanding how disease occurs with *A. phagocytophilum* is the observation that clinical signs of illness in humans and animal models are not closely linked to bacterial loads, but are more closely associated with stimulation of host immune and inflammatory signaling such as with upregulation of the genes for *interleukin 1* (*IL1*)*, C-X-C motif chemokine ligand 8* (*CXCL8*)*, matrix metalloprotease*−*9* (*MMP9*), or innate immune responses, and subversion of regulatory aspects of cytotoxic lymphocytes (6–12).

How *A. phagocytophilum* triggers or directly influences immune and inflammatory processes is of some debate, but appears tied to the ability to regulate inflammatory gene transcriptional programs, including those that operate through key transcription factors such as signal transducer and activator of transcription 1 (STAT1) or the nuclear factor kappa B (NF-κB) (8, 13–16). While it is likely that inflammatory signaling after *A. phagocytophilum* infection involves multiple pathways, as a major pathway, NF-κB signaling is likely to be central to either death or survival of the pathogen and as a mechanism by which innate immune-mediated injury occurs. Two major pathways exist for NF-κB activation, the canonical pathway chiefly leading to p50/RELA dimer transcription factor, and the non-canonical pathway, largely dependent on the generation of p52/RELB dimers. While both can be generated simultaneously, there are key differences in the specific programs incited (17–19). Canonical pathway activation is associated with classical inflammatory processes, but non-canonical activation seems to be a feature of differentiation of lymphoid cells, processing in dendritic cells, and associations with immune dysregulation and autoimmunity. Owing to the diverse clinical inflammatory manifestations of infection by *A. phagocytophilum* in humans and animals, the major objective of this study was to identify specific NF-κB signaling molecules and pathways triggered or targeted by *A. phagocytophilum* and how their alterations affect sustained bacterial growth while increasing pro-inflammatory responses of host neutrophils. Here, we examine NF-κB signaling of infected cells to determine whether *A. phagocytophilum* impacts canonical, non-canonical, or atypical NF-κB signaling. We hypothesize that inhibition or silencing of *A. phagocytophilum*-interacting pathways or genes will (i) adversely affect intracellular bacterial growth, and (ii) subsequently reverse pro-inflammatory neutrophil phenotypes. We sought to identify molecular targets that detect and signal, or at which *A. phagocytophilum* could exert influence over sustained inflammatory responses and severe disease.

## Methods

### Cell Lines and *A. phagocytophilum* Infection

The promyelocytic HL-60 (ATCC CCL-240) and myelomonocytic THP-1 (ATCC TIB-202) cell lines were purchased from American Type Culture Collection (Manassas, VA), and grown in RPMI 1640 medium (Hyclone, Thermo Fisher Scientific, Waltham, MA) supplemented with 5–10% fetal bovine serum (Thermo Fisher Scientific, Waltham, MA) and Glutamax (Life Technologies, Carlsbad, CA). All cells were grown in a humidified incubator at 37°C with 5% CO_2_. Cell density was kept <10^6^ cells/mL by diluting with fresh medium. *A. phagocytophilum* (Webster^T^ strain)-infected HL-60 or THP-1 cells were maintained as previously described (20, 21). Cell-free *A. phagocytophilum* were harvested and purified from heavily infected cells, as previously described (21), and used to infect THP-1 cells at an MOI of 100:1. For all experiments *A. phagocytophilum* was passed <10 times *in vitro*. To simulate infection over short and longer intervals of time, we used cultures at 50% (between 2 and 4 days after subculture) and 90% (4–7 days of culture) infected cells.

### Cell Fractionation, Immunoblotting, and Nuclear Proteomics Evaluation

Nuclear extracts from *A. phagocytophilum*-infected, uninfected, or LPS-stimulated HL-60 cells were prepared using NE-PER Nuclear and Cytoplasmic Extraction Reagents (Thermo Scientific/Pierce Biotechnology, Rockford, IL). Samples of the cytoplasmic and nuclear fractions were analyzed by immunoblotting for the presence of NF-κB components. Ten microgram total protein from nuclear extracts of infected and uninfected HL-60 cells was electrophoresed in 10–12% SDS-PAGE gels and transferred to nitrocellulose membranes. The membranes were blocked with 5% non-fat dried milk, probed with either NF-κB p50/p105, NF-κB p65, and IκBα rabbit polyclonal antibodies (Santa Cruz Biotechnology) and incubated with goat anti-rabbit IgG or anti-mouse IgM alkaline phosphatase conjugate (KPL, Gaithersburg, MD); loading controls were not used for these initial studies. The immunoblots were visualized by incubation with BCIP/NBT substrate (Bio-Rad, USA), and the migration of the specific bands was compared with that of Jurkat nuclear extract positive control (Santa Cruz Biotechnology) or with nuclear protein isolated from LPS-stimulated HL-60 cells. These experiments were repeated 3 times.

We further examined data extracted from a proteomics (iTRAQ [isobaric tag for relative and absolute quantitation protein profiling technology; Applied Biosystems]) study using *A. phagocytophilum*-infected and uninfected cells HL-60 cells that identified nuclear proteins in infected and uninfected HL-60 cells and their ratios (22). For each protein identified, two types of scores were reported: unused ProtScore and total ProtScore. The total ProtScore measures all peptide evidence for a protein analogous to protein scores reported by other protein identification software programs. However, the unused ProtScore measures all peptide evidence for a protein not better explained by a higher ranking protein, and was the method of choice. The protein confidence threshold cutoff for this study was set at an unused score of 2.0 with at least one peptide with 99% confidence. A ratio of infected to uninfected (Aph:HL-60) score was used to identify differential presence of human proteins in nuclear lysates. To do this, we averaged the ratios of uninfected HL-60 nuclear lysate replicates (isobaric isotope labels 115:114) and ratios of nuclear lysate replicates from *A. phagocytophilum*-infected HL-60 cells (116:114 and 117:114) to create the composite Aph:HL-60 mean ratio. Proteins identified with mean ratios (fold change infected/uninfected) > 2 or < 0.5 were considered to be significantly differentially present in the nucleus. This experiment was done once with biological replicates.

Finally, we compared gene expression profiles among 4 separate studies that examined *A. phagocytophilum* infection in neutrophils (GSE2405; GPL570) (8, 14) HL-60 cells, all trans-retinoic acid (ATRA)-differentiated HL-60 cells (23) (GSE107770), and NB4 promyelocytic cells (24) (GSE2600) to identify differential expression of 104 genes associated with NF-κB signaling in the KEGG (Kyoto Encyclopedia of Genes and Genomes) database (https://www.genome.jp/dbget-bin/www_bget?pathway+hsa04064). This pathway is roughly divided into “canonical,” “atypical,” and” non-canonical.” The downloaded list was manually annotated for each protein/gene with regard to the 3 pathways in which each participates for signaling. The set was examined for consensus differential transcript in two of the three studies examined, with up- and downregulation considered as per authors, in all 3 cases, a 2-fold change compared to control uninfected neutrophils or HL-60 cells; *p*-values or false discovery rate were collected as available (Supplementary Table 1C).

### Inhibitors and Toxicity Studies

In order to determine which of the NF-κB activation pathways was utilized by *A. phagocytophilum*, we selected specific pharmacologic inhibitors that could differentially block either (i) the proteasome, using MG132 (N-[(Phenylmethoxy)carbonyl]-L-leucyl-N-[(1S)-1-formyl-3-methylbutyl]-L-leucinamide); (ii) the canonical pathway at NF-κB essential modulator/inhibitor of nuclear factor kappa B kinase regulatory subunit gamma (NEMO/IKKγ), using NEMO-Binding Domain Binding Peptide (NBD peptide; DRQIKIWFQNRRMKWKKTALDWSWLQTE, and control peptide DRQIKIWFQNRRMKWKK-TALDASALQTE); (iii) the non-canonical pathway using either wedelolactone (1,8,9-Trihydroxy-3-methoxy-6H-benzofuro[3,2-c][1]benzopyran-6-one, 7-Methoxy-5,11,12-trihydroxycoumestan; an inhibitor of both inhibitor of nuclear factor kappa-B kinase subunit alpha [IKKα,IKK1,CHUK]) and nuclear factor kappa-B kinase subunit beta [IKKβ,IKK2] (25) or SC 514 (4-Amino-[2′,3′-bithiophene]-5-carboxamide; a selective ATP-competitive inhibitor of IKKβ (26); or (iv) the atypical pathway (inhibitor of nuclear factor kappa-B kinase [IKK]-independent) using Casein Kinase II Inhibitor I (CK2i; 4,5,6,7-Tetrabromobenzotriazole), a high affinity, cell-permeable inhibitor of casein kinase 2 (CK2) (all from Millipore/Sigma, USA). To implement these studies, we selected to use the myelomonocytic cell line THP-1, owing to its ready ability for manipulation and transfection. Initially, all drugs were tested over the range of 12.5, 25.0, and 50.0 μM concentrations to assess both THP-1 and *A. phagocytophilum*-infected THP-1 cell (4 × 10^5^ cells) toxicity using the ToxiLight Bioassay Kit (Lonza) compared to cells treated with 1% Triton X and untreated cells. Percent cytotoxicity was calculated (luminescence units of cells with or without drug/luminescence units of cells treated with Triton X) and then normalized to untreated cells. Normalized values >2 (50% cytotoxicity) were considered toxic.

### Quantitative Measurement of *A. phagocytophilum* Growth and Inhibitor Impact on Defense Gene Transcription

*A. phagocytophilum* was quantified by three methods: (1) cytocentrifugation of infected THP-1 cells, followed by Romanowsky staining (LeukoStat) and enumeration of the proportion of infected cells (repeated 3 times); (2) quantitative real-time 5′-nuclease PCR, targeting *A. phagocytophilum msp2* DNA, as previously described (27, 28) (repeated 3 times in triplicate); and (3) quantitative reverse transcriptase PCR, targeting *A. phagocytophilum major surface protein-2* (*msp2*) mRNA using the same 5′ nuclease method (repeated twice in triplicate). Quantification of PCR assays was accomplished using the BioRad CFX384 Real time PCR analyzer, by comparing mean Cq values of known concentrations of cloned *A. phagocytophilum msp2* as a single copy in a plasmid, and dividing by a factor of 84 (the approximate number of *msp2* paralogs detectable by this assay in the *A. phagocytophilum* Webster strain genome) for DNA. All samples were tested in at least duplicate.

To determine whether specific pharmacologic inhibition of pathways in the canonical, non-canonical, or the “atypical” IKK-independent pathway would impact infection, growth or survival of *A. phagocytophilum*, infected THP-1 cells were pretreated with a range of non-toxic doses of drugs targeting each pathway, infected with cell-free *A. phagocytophilum* at an MOI of 100:1, and examined after 24 h of growth *in vitro* by qPCR or after 6 days of growth for microscopic examination, comparing untreated to treated cells. Similarly, cells were harvested from triplicate cultures for preparation of RNA and analysis of relative transcription of cellular *CXCL8* and *MMP9*, markers of κB-driven pro-inflammatory gene regulation with *A. phagocytophilum* infection. Transcriptional responses were normalized (ΔCt) to housekeeping genes for infected and uninfected cells separately, then to *CXCL8* and *MMP9* transcription (ΔΔCt) for no inhibitor vs. inhibitor in infected and uninfected cells separately. To extend the findings of pharmacologic inhibitors of NF-κB signaling genes, similar studies were conducted in a high-throughput screen using 96 distinct target genes, including 92 related to NF-κB signaling (Supplementary Table 1A) and 4 housekeeping genes previously shown to be largely unaffected by *A. phagocytophilum* infection (*ACTB, B2M, GAPDH*, and *HPRT1*) (29, 30). Assays were conducted as above in smaller scales comparing uninfected THP-1 cell gene transcription to that of *A. phagocytophilum*-infected THP-1 cells with or without 25 μM SC-514 or wedelolactone. Transcription for each of the 96 genes was measured by preparing RNA from duplicate cultures after 24 h of infection and testing each culture in technical replicates, measuring increase in SYBR green fluorescence at the Ct values for each culture and technical replicate. Final values for relative transcription were calculated after averaging the housekeeping gene expression Ct values to calculate initial ΔCt values (housekeeping gene Ct—target gene Ct), and using the ΔΔCt method by comparing ΔCt expression values of all variables to ΔCt values for uninfected or infected cells (21). In general, a 2-fold increase or decrease in transcription was considered significant. However, statistical analysis was conducted by comparing transcription in replicate cultures using the Student's *t*-test with and without the Benjamini-Hochberg method for False Discovery rate (FDR) correction (http://www.real-statistics.com/). FDR < 0.05 was considered significant. These experiments were done twice.

### NF-κB Signaling Pathway Gene Silencing

To determine the impact of genes involved in NF-κB signaling on the propagation of *A. phagocytophilum*, we used the SureSilencing™ siRNA Array Human NF-κB Signaling Pathway- Validated Gene Knockdown RNA Interference kit (SABiosciences/Qiagen), according to manufacturer instructions. Here, 6.4 × 10^3^ to 1.28 × 10^4^ THP-1 cells were aliquoted into the respective wells after reagent rehydration with SureFECT Transfection Reagent in medium and allowed to become transfected for 12 h. Thereafter, cell-free *A. phagocytophilum* was added at an MOI of 200:1 (2 × 10^6^ bacteria) and incubated for 6 h. This MOI reproducibly yielded a significant and discernable increase in bacterial quantity within 6 h in pilot experiments (not shown). At 18 h, samples were removed for microscopy and quantitation of infected cells; the remainder was used for RNA preparation and reverse transcriptase PCR. Relative gene transcription was evaluated by SYBR green reverse transcriptase PCR targeting mRNA transcripts included for silencing in the SureSilencing™ siRNA Array Human NF-κB Signaling Pathway kit; *GAPDH* and *HRPT1* were used as housekeeping genes. Melt curve analysis was used to assure specificity of the SYBR green PCR reactions. Silencing efficiency was evaluated as per manufacturer's instructions for THP-1 cells, and only those targets for which significant reduction of at least 75% expression was observed were evaluated for changes in *A. phagocytophilum* RNA quantity as an estimate of bacterial viability and propagation in gene-silenced cells; data for selected gene targets with >55% reduction in expression were also evaluated. *A. phagocytophilum* growth or suppression was monitored by simultaneous reverse transcriptase amplification of *msp2* transcripts within the RNA preparations, using the factor of 119 (number of *msp2* RNA transcripts for each *msp2* genome equivalent) to convert to genome equivalents based on comparative RNA and DNA PCR using known quantities of bacteria after 18–24 h active replication (data not shown). This experiment was done twice.

## Results

### Prolonged Nuclear Localization of NF-κB in *A. phagocytophilum*-Infected HL-60 Cells

**Figure 1:**
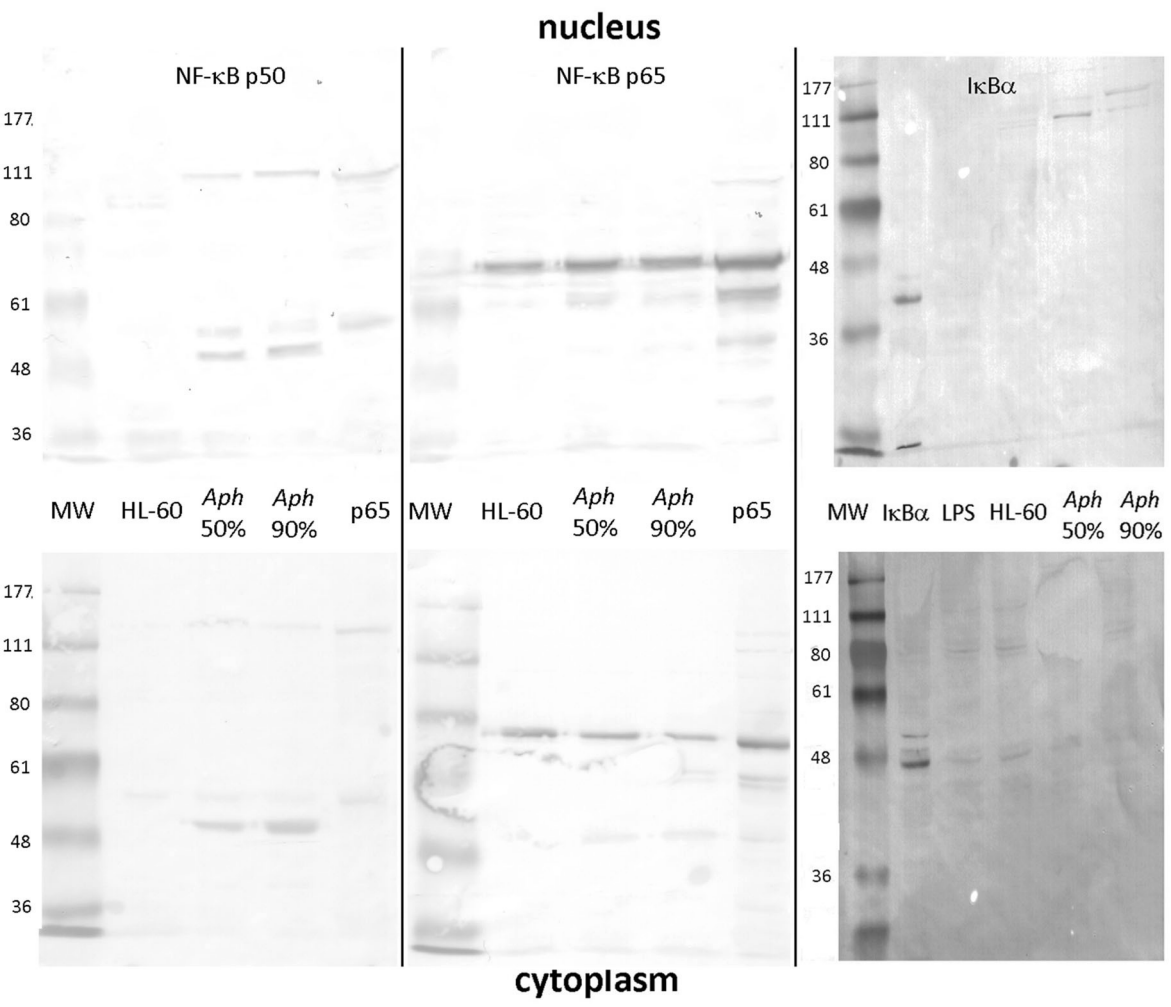
NF-κB p50/p105, RELA (p65), and IκB analysis in the nucleus and cytoplasm of *A. phagocytophilum*-infected and uninfected HL-60 cells. The p50 component of p105 is expressed as two isoforms in infected HL-60 cells, and qualitatively lower density in uninfected than in infected cells; p50 is also detected as its precursor, p105 in both nucleus and cytoplasm (left panels). Unlike p50 and p105, RELA (p65) is qualitatively similar in uninfected and infected HL-60 cell nuclei and cytoplasm (middle panel). IκBα is also detected qualitatively at very low band density in the cytoplasm but not nuclei of uninfected, LPS-stimulated, and infected HL-60 cells as monomers; it is also detected in the nuclei of infected HL-60 cells but only as high molecular weight complexes, likely reflecting ubiquitylation. The antibody used to detect bands for each vertical nuclear (top panels) and cytoplasm (bottom panels) extract pair is labeled at the top. Jurkat cell nuclear lysate positive control = pos. The image represents a composite of six separate gel images, altered only for decreased brightness to visualize all bands, applied equally to all panels. No other manipulations were used.

As shown in Figure 1, uninfected HL-60 cells demonstrated presence of RELA p65 but lacked detectable NF-κB p50 (and precursor p105) in nuclei and cytoplasm. In contrast, infection of HL-60 cells of sufficient duration to achieve 50% infected cells (~2–4 days) or 90% infected cells (~4–7 days) demonstrated the presence of both nuclear p65 and the presence and persistence of p50 in infected cell nuclei. While p50 was detected also as a higher molecular weight mobility, likely as precursor p105, p65 was detected predominantly as a monomer, with possible isoforms or post-translational modifications in both the nucleus and cytoplasm (31). Similarly, IκBα was marginally detectable as a 36–41 kDa monomer in the cytoplasm of uninfected, infected, and LPS-stimulated HL-60 cell; however, in the nucleus, IκBα was not detected in either uninfected or LPS-stimulated HL-60 cells, but was present within high molecular weight bands at ~100 to 150 kDa among *A. phagocytophilum*-infected cells, likely reflecting ubiquitylation (32, 33).

Using the iTRAQ proteomics approach, infected HL-60 cells demonstrated 848 human nuclear protein signatures, of which 26 were present in 2-fold greater or lesser amounts than in uninfected cells; of note, NF-κB2 was identified expressed 2.5-fold higher than in uninfected cell nuclei, but among 11 NF-κB signaling signatures (Supplementary Table 1B) detected in the nuclear preparations, 10 were not differentially expressed >2-fold. IκBα, the inhibitor of NF-κB in the cytoplasm and nucleus (33, 34), was not identified, perhaps owing to stimulation of the canonical pathway leading to its ubiquitylation and degradation. When transcriptional profiles of *A. phagocytophilum*-infected myeloid cells were queried for differential expression of a set of 104 NF-κB signaling pathway genes, 9 were upregulated >2-fold and none were downregulated more than 2-fold in 3 of 4 datasets evaluated (Supplementary Table 1C).

### Toxicity of Pharmacologic Reagents

THP-1 cells, infected or not by *A. phagocytophilum*, generally tolerated up to 25 to 50 μg/mL of NF-κB inhibitors, except MG-132, which was cytotoxic in all concentrations tested for uninfected, but not infected cells (Supplementary Figure 1), and NBD peptide, which was toxic at concentrations >12.5 μM in uninfected cells (Supplementary Figure 1); NBD peptide was not further tested. The optimal concentrations of each inhibitor were determined by the highest concentration of the inhibitor that was not cytotoxic for uninfected or infected cells. In general, each drug was tested over the range of concentrations including 12.5, 25.0, and of 50.0 μM.

### Effects of Pharmacologic Inhibitors of NF-κB Signaling on *A. phagocytophilum* Infection and Growth

**Figure 2:**
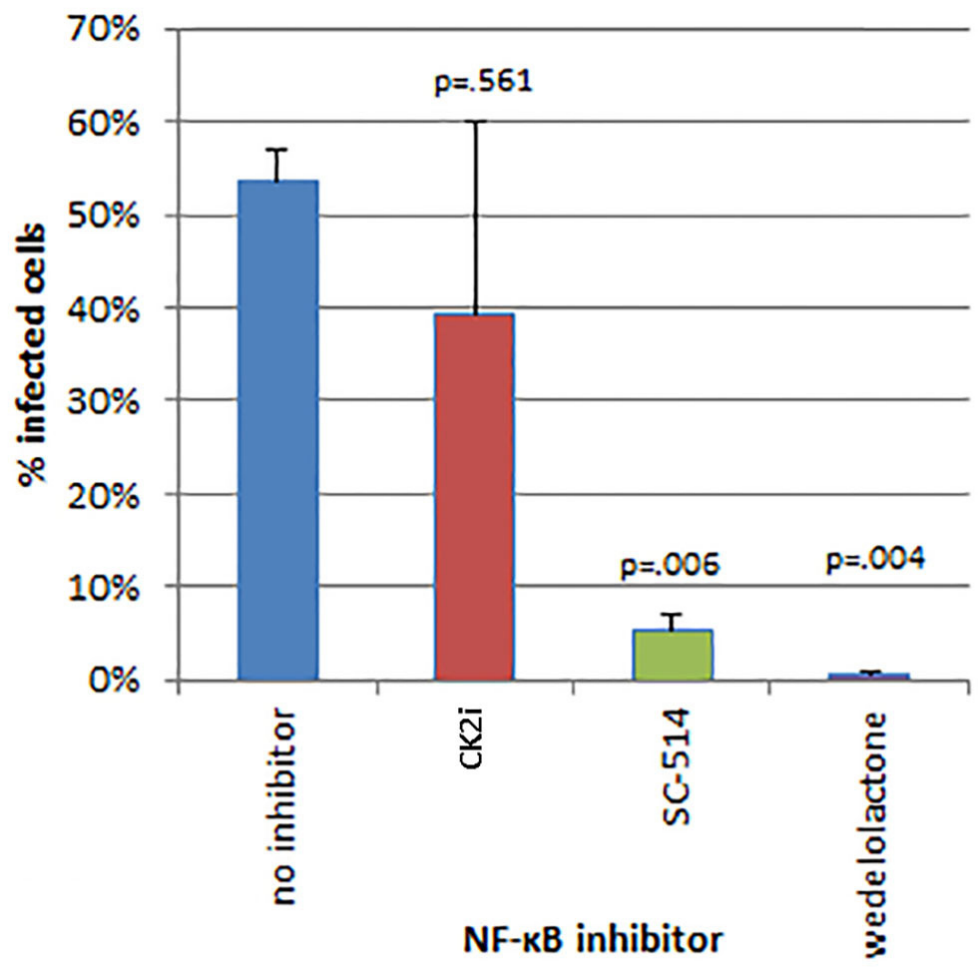
Inhibitors of the alternative or non-canonical NF-κB signaling pathway (wedelolactone—inhibitor of IKKα/β; SC-514—selective inhibitor of IKKβ) significantly inhibit *A. phagocytophilum* growth when examined microscopically for the presence of morulae (intravacuolar bacterial colonies). The casein kinase 2 inhibitor of the IKK-independent pathway (CK2i), did not significantly affect bacterial growth. Error bars are SEM; *p*-values were calculated comparing to no inhibitor.

**Figure 3:**
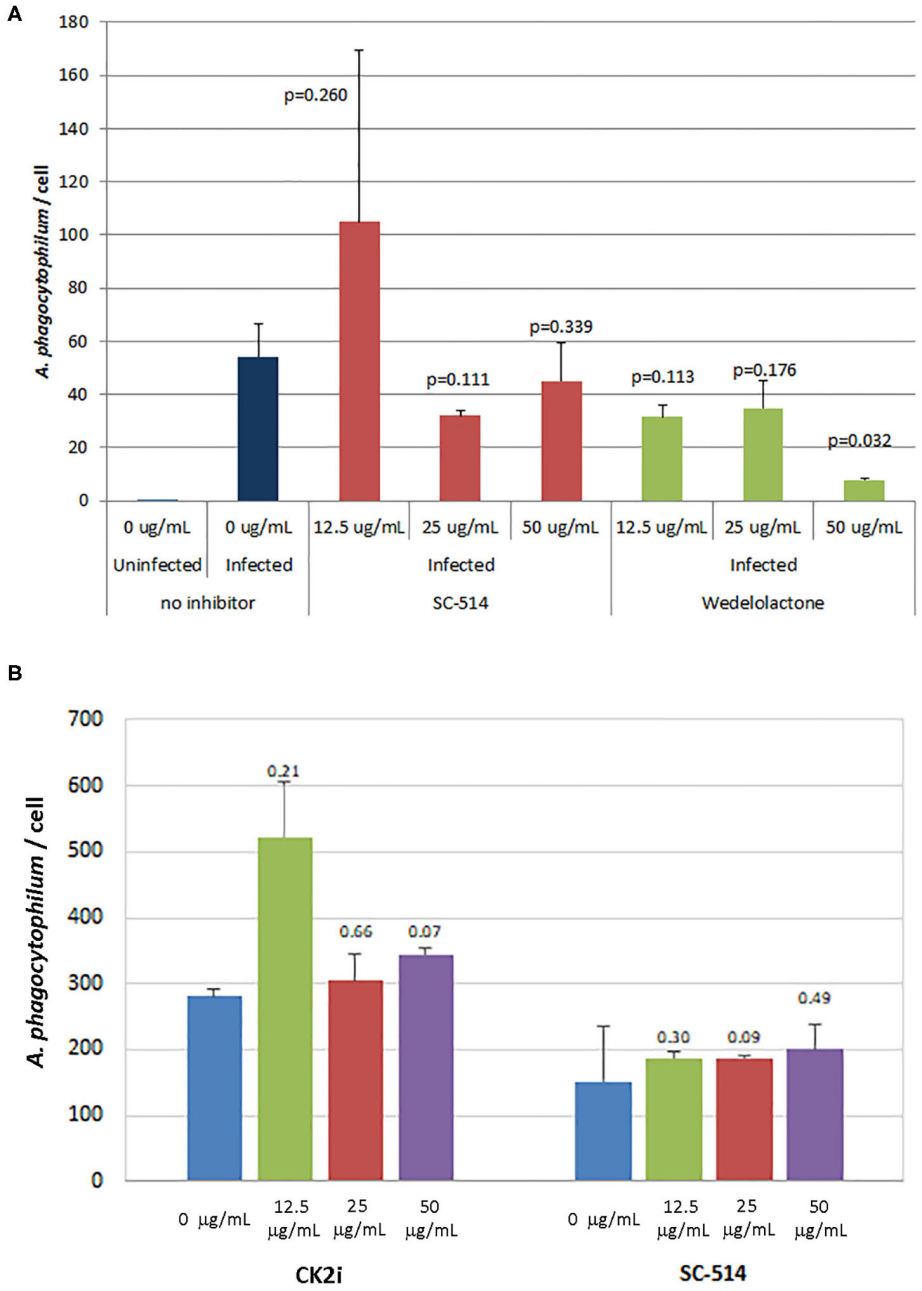
Wedelolactone, a non-selective IKKα/β inhibitor of the alternative or non-canonical NF-κB signaling pathway **(A)**, but not SC-514 that selectively inhibits IKKβ in the non-canonical pathway **(A,B)**, or CK2i, and inhibitor of the IKK-independent pathway **(B)**, significantly inhibits *A. phagocytophilum* growth when used in high concentrations and when assessed by quantitative PCR. Error bars are SEM; *p*-values were calculated comparing to no inhibitor and are listed above each average measurement.

The effects of inhibitors of NF-κB signaling on *A. phagocytophilum* infection and growth was greatest for wedelolactone and to a lesser degree for SC-514, with no effect for CK2i. The impact of inhibition of the non-canonical pathway by wedelolactone and SC-514 was most dramatic in the microscopic evaluations (Figure 2) where growth was reduced between 99 and 96% (*p* = 0.035 and 0.015, respectively). In contrast, growth was not significantly different compared to no drug for CK2i that impacts the IKK-independent pathway. The proteasome inhibitor, MG-132, which targets both the canonical and non-canonical NF-κB pathways, was cytotoxic in the high drug concentration used for the microscopic studies. Because of this limitation, *A. phagocytophilum* growth was also assessed by qPCR using pharmacologic inhibitors over a range of drug doses shown to lack cytotoxicity. In general, bacterial quantity/cell was lower with wedelolactone at high concentrations (Figure 3). Unlike the observations by microscopy, SC-514 had no significant impact on *A. phagocytophilum* growth at any dosage. Triplicate experiments with the CK2 inhibitor CK2i revealed no significant changes in *A. phagocytophilum* growth for any of the 3 concentrations tested.

**Figure 4:**
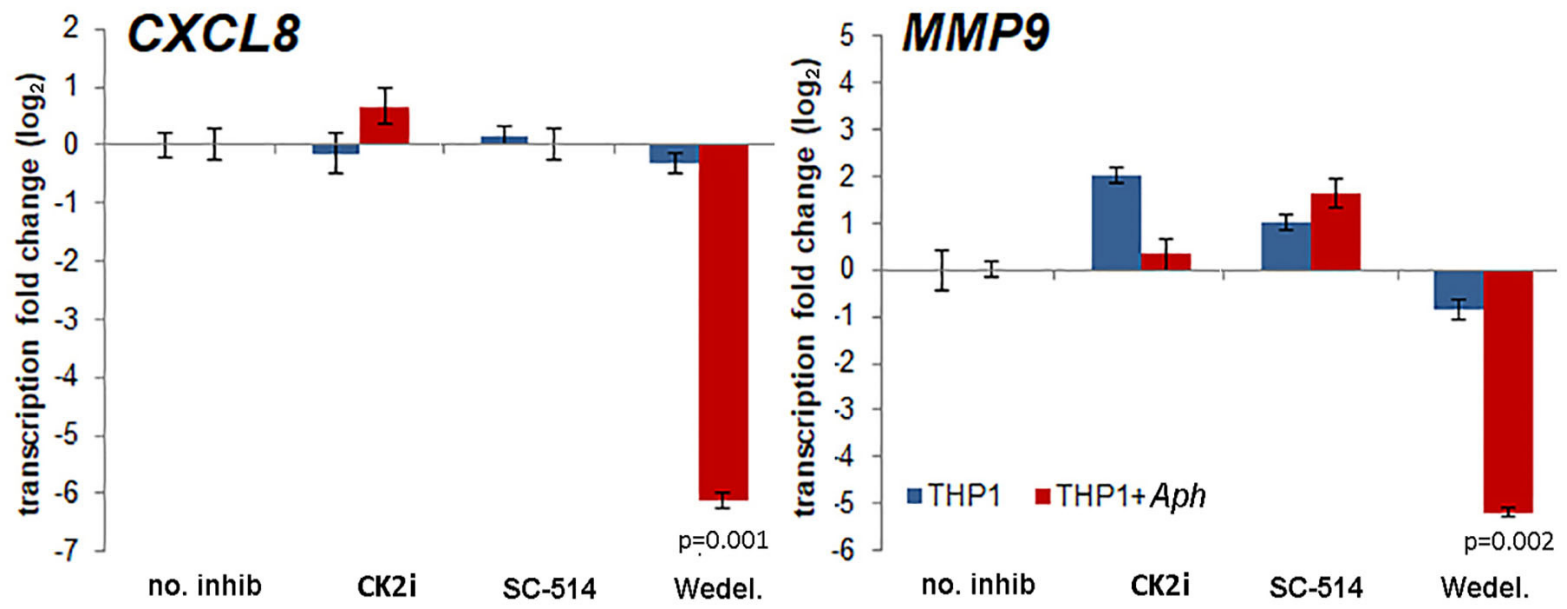
Activation of the non-canonical NF-κB signaling pathway mediates upregulation of downstream gene expression important for *A. phagocytophilum* immune pathogenicity. Differential transcription from *CXCL8* encoding the key chemokine that recruits new host neutrophils, and from *MMP9* encoding an important protease in neutrophils that is secreted as part of the inflammatory process after *A. phagocytophilum* infection, are significantly dampened by the non-selective IKKα/β inhibitor, wedelolactone (Wedel.), but not by other inhibitors of NF-κB signaling, and a similar effect was not present with uninfected cells. Error bars represent SEM; *p*-values infected vs. uninfected THP-1 are shown when <0.05.

The effects of pharmacologic inhibition of NF-κB signaling inhibitors on downstream expression of *CXCL8* and *MMP9*, genes known to be upregulated with and important for *A. phagocytophilum* pathogenesis (6–8, 23, 35), were evaluated. Increasing concentrations of the NF-κB signaling pathway inhibitors had marginal reproducible effects for all drugs tested in infected cells, with the exception of wedelolactone, for which concentrations higher than 12.5 μM significantly diminished *CXCL8* transcription, and also dampened transcription of *MMP9*. *CXCL8* transcription was also significantly reduced in uninfected THP-1 cells with all doses of wedelolactone. When confirmed with optimized doses of drug based on initial experiments (Wedelolactone 31.8 μM, SC-514 50 μM, and CK2i 20 μM), the silencing of *CXCL8* and *MMP9* was greater among infected cells (~100-fold reduced) than uninfected cells (Figure 4), with significant reductions only observed for wedelolactone (*p* = 0.001 to 0.002).

**Figure 5:**
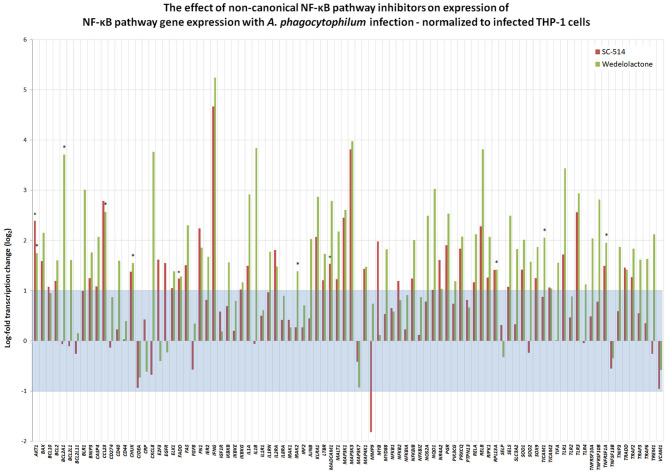
Inhibition of non-canonical NF-κB activation with wedelolactone or SC-514 reverses suppression of many genes transcriptionally dampened with *A. phagocytophilum* infection (see Supplementary Figure 2). Here, gene expression was normalized to that observed in *A. phagocytophilum*-infected THP-1 cells. Beyond the borders of the blue zone depicts >2-fold change compared to transcription in infected cells without the pharmacologic inhibitors. The most highly impacted genes were all upregulated by either wedelolactone (IKKα/β inhibitor) or SC-514 (IKKβ inhibitor) and included *AKT1, FADD, MADCAM11, MAP3K1, BCL2A1, CCL23, CHUK, IRAK2, RPL13A, TICAM1, TNFRSF1A*, and *TNFRSF10A*. Values that represent a significant difference (*p* < 0.05) compared to infected cells are denoted by an asterisk “*”.

To discern whether *A. phagocytophilum* also influences the transcription of genes involved in NF-κB signaling, and whether this could be impacted by pharmacologic inhibition by either Wedelolactone or SC-514, RNA from uninfected, and *A. phagocytophilum*-infected THP-1 cells with our without 25 μM wedelolactone or 25 μM SC-514 were assayed by RT-PCR. A majority (64/92) of NF-κB signaling pathway genes were downregulated more than 2-fold with infection, whereas only 5 of 92 were upregulated, *BCL2A1, IL1B, CXCL8, MMP9*, and *SOD2* (Supplementary Figure 2). Eight of 92 genes were downregulated more than 6.25-fold (*CCL23, FGF8, IFNG, MADCAM1, MAP3K3, PGR, PRKCG*, and *PTPN13*); only *BCL2A1* had a *p* < 0.05, and no gene expression was significantly changed when analyzed by the Benjamini-Hochberg method for False Discovery rate (FDR) (Supplementary Figure 2). When normalized to uninfected cells, of the 92 genes assayed in infected cells treated with SC-514, 24 were upregulated and 11 downregulated more than 2-fold, but only 4 achieved unadjusted *p* < 0.05 (*AKT1, FADD, MADCAM1*, and *MAP3K1*), and none were significant when adjusted for FDR. Wedelolactone had more dramatic effects, increasing expression of 15 and decreasing expression of 23 out of the 92 genes examined. Of these, only 10 showed upregulated expression with unadjusted *p* < 0.05, including *AKTA, BCL2A1, CCL23, CHUK, IRAK2, MAP3K1, RPL13A, TICAM1, TNFRSF10B*, and *TNFRSF1A*, but only *RPL13A* (*p* = 0.0144) and *TICAM1* (*p* = 0.0058) were significantly upregulated compared to infected cells after adjustment for FDR (Figure 5). It should be noted that *CXCL8* and *MMP9* that were downregulated in infected cells treated with wedelolactone, but not SC-514 (Figure 4), were not significantly changed by similar inhibitor treatments in the high-throughput study. This observation could be an effect of the differing assay sensitivities, uncontrolled biological variables over time that perhaps lead to differential activation of canonical and non-canonical pathways in the same experiments, all potentially confounding interpretations.

### NF-κB Signaling Pathway Gene Silencing

**Figure 6:**
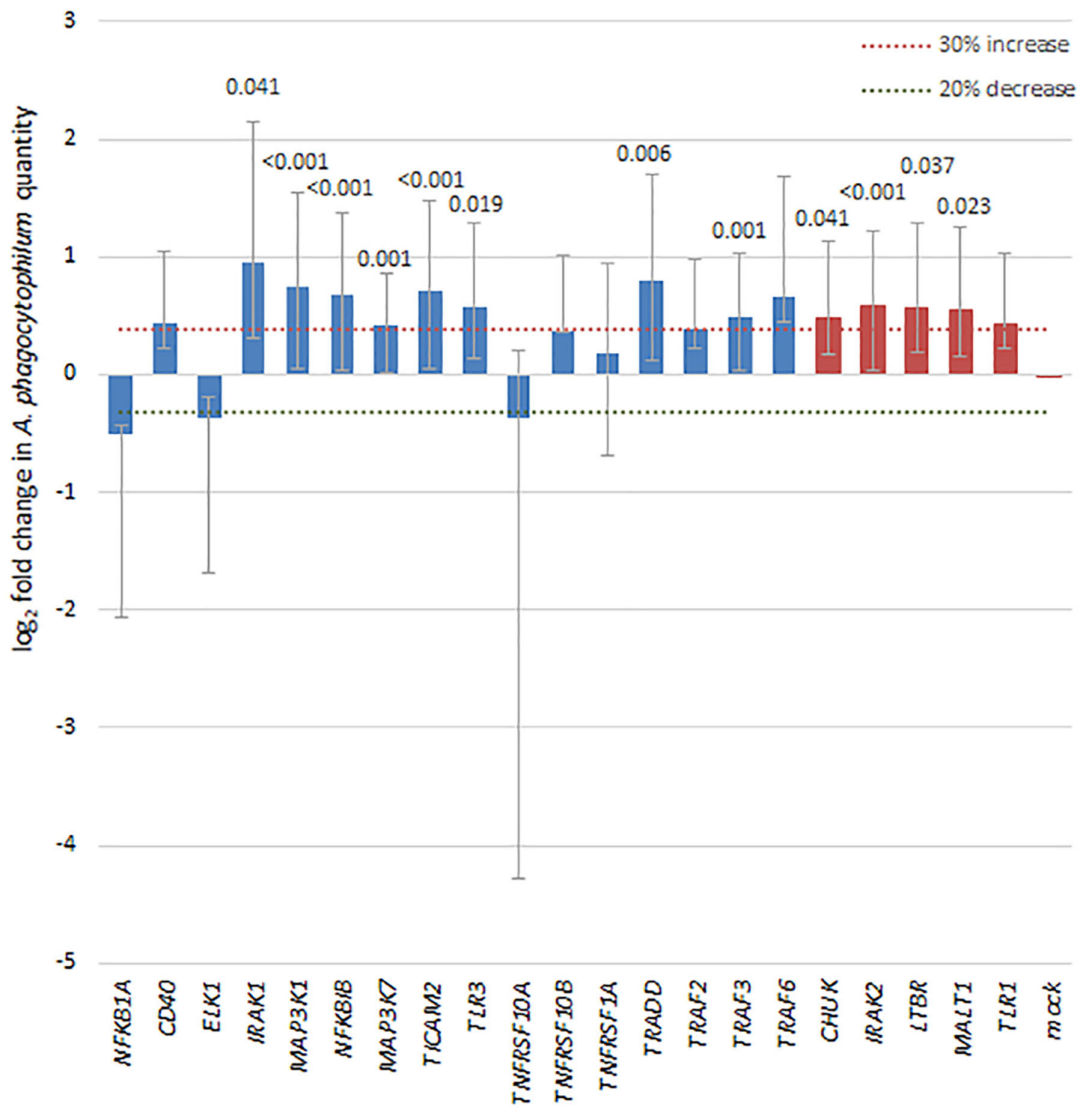
Silencing of NF-κB signaling pathway genes by siRNA leads to a 30% or greater increase in *A. phagocytophilum* growth, as measured by qPCR in 8 of 16 genes with silencing >75% (blue bars), including *IRAK1* (canonical)*, MAP3K1* (canonical)*, NFKBIB* (both)*, TLR3* (both)*, TRADD* (canonical)*, TRAF3* (both), and *MAP3K7* (canonical), involved largely with the canonical pathway via TNF receptor or TLR signaling pathways. In addition, among 5 other genes silenced to at least 55% (red bars), 4 (*CHUK, IRAK2, LTBR*, and *MALT1*) also demonstrated significant increases in *A. phagocytophilum* growth. Error bars represent SEM; when significantly different from the mock-silenced control (*p* < 0.05), *p*-values are provided above each bar.

To discern whether broader effects of *A. phagocytophilum* infection resulted in transcriptional regulation of a variety of genes involved in NF-κB signaling, a transcriptional profiling array was employed to analyze the effects of infection in silenced THP-1 cells vs. effects in cells with control siRNA. Of the 41 NF-κB signaling pathway genes targeted in the SureSilencing™ siRNA Array kit (Supplementary Table 2), 16 genes were silenced by 75% or greater. Among the NF-κB signaling pathway genes successfully silenced in THP-1 cells, *A. phagocytophilum* levels increased by more than 30% and with *p* < 0.05 with silencing of *TRADD, TRAF3, MAP3K1, MAP3K7, IRAK1, TLR3, TICAM2*, and *NFKB1*, and of 4 with growth decreased by 20% or more, none were significantly different than mock silenced cells (Figure 6). Five additional genes silenced >55% were also considered, and 4 of these demonstrated a significant increase in *A. phagocytophilum* growth as compared with mock-silenced controls; these included *CHUK* (*IKKA*)*, IRAK2, LTBR*, and *MALT1*. Their inclusion allowed an improved analysis of the non-canonical pathway.

## Discussion

*A. phagocytophilum* has a remarkable capacity to survive within the harsh niche of the primary innate immune antimicrobial cell, the neutrophil. It does this by virtue of altering fundamental functions of this cell, including reprogramming of (i) pro-inflammatory responses; (ii) apoptosis; (iii) antimicrobial responses; and (iv) reduction of tethering/arrest and emigration capacity. The overall fitness benefit to the bacterium is (i) pro-inflammatory responses and recruitment of new host neutrophils into which it can pass and propagate (6, 10, 15, 16, 35, 36); (ii) dampening of mechanisms that could lead to bacterial killing within the neutrophil (21, 30, 37–41); (iii) prolongation of neutrophil survival through delayed apoptosis to permit increased bacterial doubling (8, 42–44); and (iv) increased retention of infected cells within the vasculature accessible for transmission during a subsequent tick blood meal (7, 45–49). Each of these distinct functions has been examined to some degree and many depend intrinsically on host gene expression and transcriptional programs, modulation of signaling pathways, or subversion of intracellular trafficking, some proven to be regulated by microbial manipulation of host gene transcription (21, 30, 41). While it would be anticipated that *A. phagocytophilum* should initiate pro-inflammatory responses via interactions with toll-like receptors and other microbial sensors, coordination of host inflammation ordinarily results in parallel activation of both inflammatory and antimicrobial activities as well as the ability of activated cells to transmigrate microvascular barriers in response to chemotactic gradients. Moreover, the lack of most differential transcription elicited when cells are stimulated by heat-killed *A. phagocytophilum* (8), suggests that active infection and host cell manipulation are key to not only pathogen survival, but to pro-inflammatory responses. These events are coordinated by a number of mechanisms; but most converge with activation and nuclear translocation of NF-κB, and binding of its active components to κB consensus sequences over a spectrum of gene promoters that execute the neutrophil's inflammatory, survival, and antimicrobial activities.

An overriding concept in intracellular infections is that pathogens modulate host signal transduction and transcriptional responses to change host cell function and improve bacterial survival. Intracellular pathogens that alter host signaling are best studied in macrophages and epithelial cells, while little is known about bacteria that propagate within neutrophils. In macrophages, bacterial proteins introduced into host cells influence phagocytosis, vacuolar trafficking, and promote or dampen inflammation, apoptosis and cytotoxicity, among other effects. One strategy targets signal transduction of important pro-inflammatory pathways, particularly by interfering with activation of NF-κB (50). The full spectrum of NF-κB-responsive genes has not been described, but important targets include those involved in innate and adaptive immunity, such as genes for cytokines, chemokines, adhesion molecules, acute phase proteins (SAA), inducible effectors (iNOS, COX-2), adaptive immune response, and regulators of apoptosis and cell proliferation (51), many of which comprise the transcriptional reprogramming phenotype of *A. phagocytophilum*-infected neutrophils. NF-κB is a target for bacterial and viral subversion of host cells (50), where, for example, *Yersinia enterocolitica* YopP binds to IKKβ, the kinase that phosphorylates IκBα before proteolysis and release of activated NF-κB (52). Likewise, *Mycobacterium ulcerans* affects NF-κB transactivation by preventing nuclear localization or by interfering with DNA binding (53), and NF-κB can be regulated by viral IκBα mimics suppressing inflammatory response (54).

NF-κB/REL proteins comprise a family of dimeric transcription factors, including RELA (p65), RELB, c-REL, NF-κB1 p50 (and p105 precursor), and NF-κB2 p49/52 (and p100 precursor) (51). Most abundant is the p50/p65 heterodimer that rapidly transactivates genes for transcription; p50/p50 and p52/p52 homodimers can repress target gene transcription. NF-κB/REL proteins possess a conserved N-terminal Rel homology domain (RHD) that mediates DNA binding, dimerization, and interaction with IκB inhibitory proteins, to which they are bound in an inactive state in the cytoplasm. The RHD also has nuclear localization sequences (NLS) usually masked by interaction with IκBs. IκBs comprise a family with several members, IκBα, IκBβ, IκBε, IκBγ, Bcl-3, and the Rel precursors, p100 and p105. Control of NF-κB gene transactivation is based on phosphorylation of IκBs at specific sites that targets them for ubiquitylation and proteasome degradation, unmasking the NLS on NF-κB to allow transport into the nucleus where transcriptional effects are mediated (51).

NF-κB activation is affected by a number of signals including receptors such as toll-like (TLR) or tumor necrosis factor (TNFR) receptors, and is propagated by signaling through MyD88-dependent or -independent pathways resulting in IKK complex activation and eventual phosphorylation of IκBα in the canonical pathway (55, 56). The alternative or non-canonical pathway to NF-κB activation, particularly for p49/52 (NF-κB2) and RELB, results from stimuli through TNFR family members, such as CD40, lymphotoxin-β receptor, or those for LPS, via NIK (MAP3K14) upregulation and activation of IKKα or via receptor-induced degradation of TRAF2 and TRAF3 that under steady state conditions degrade NIK (18, 57). Additional activation of NF-κB occurs through intracellular interactions with NOD1 and NOD2, but whether this involves IKK complex activation is unclear (58–61). Although NF-κB activation occasionally occurs without IKK complex activation, activation of the IKK complex is often the key to NF-κB activation. A key distinction between canonical and non-canonical activation is the sole utilization of IKKα dimers in the non-canonical pathway, allowing their distinction by comparing the effects of the IKK-non-selective inhibitor wedelolactone to those of the IKKβ-selective inhibitor SC-514. This unique pharmacologic inhibition would predict that more dramatic effects with wedelolactone than for SC-514 are the result of non-canonical pathway inhibition. It is obvious that *A. phagocytophilum*, which benefits when inflammation is modulated, is provided ample targets for pathogen effectors in NF-κB signaling pathways.

Here, we demonstrate that both p65 and p50 are present in the nuclei of *A. phagocytophilum*-infected cells for at least 7 days of infection, although p65 is constitutively expressed in uninfected HL-60 cells (62). The presence of NF-κB p105 in nuclear lysates was not anticipated, but is well-documented with stimulation (63–65); whether it would provide IκB-like inhibitory function in this circumstance is not known, but likely. While neutrophils when stimulated with LPS or TNF will demonstrate nuclear p50 and p65 over periods that persist for 12 h or longer, this is in part controlled by the simultaneous nuclear retention of IκBα (33, 34), and ultimately such neutrophils become apoptotic owing to the inhibition of κB site transcriptional activation with prolonged IκBα binding (33, 34). Additionally, extrapolation from THP-1 or HL-60 cells to neutrophils is challenging; however, *A. phagocytophilum* can delay apoptosis in infected neutrophils for as long as 96 h via mechanisms active in MAP kinase signaling, sustained *BCL2* gene family transcription, and through mitochondrial stabilization (43, 44, 66). In contrast to our hypothesis, silencing of NF-κB signaling genes and inhibition of their protein products, when effective, largely increased bacterial propagation suggesting that their presence with activated NF-κB signaling lowers the fitness of *A. phagocytophilum*. For 8 of these silenced genes, *A. phagocytophilum* burden increased 30% or greater, including with silencing of *TRAF3* and *TRADD*, confirming that the canonical NF-κB signaling pathway is impacted by *A. phagocytophilum* infection. The major unexplained discrepancy for this finding is the diminished transcription of both *CXCL8* and *MMP9* in infected cells treated with the non-selective IKK inhibitor wedelolactone. This observation is of interest since CXCL8 and MMP9 are implicated as important for *A. phagocytophilum* fitness by enhancing bacterial expansion *in vitro* and *in vivo* (6, 7, 35, 67).

In contrast with the concurrent increase of RELA (p65), NFKB1 p50 and IκBα in the nuclei of activated or infected HL-60 cells and evidence that the canonical NF-κB pathway is active, is the increased detection of a NF-κB2 protein in infected vs. uninfected HL-60 cell nuclei as long as 7 days after infection, and increases in *NFKB2* transcription among transcriptional profiling studies (Supplementary Table 1C). Inhibition of *A. phagocytophilum* propagation and differential expression of κB-driven genes by the non-selective IKKα/β inhibitor wedelolactone and less robustly by the IKKβ inhibitor SC-514 was reproducible. Because signaling for the non-canonical pathway depends predominantly on IKKα, which selectively phosphorylates p100 when associated with RELB (68), the more consistent impact of wedelolactone than the IKKβ-selective inhibitor SC-514 further supports a role for signaling through the non-canonical pathway.

The non-canonical pathway is typically linked to stimulation of cell surface receptors such as the TNF receptor family CD40 (TNFRSF5) or RANK (TNFRSF11A), via lymphotoxin-β (LTB) or the B-cell TNF-family cytokine BAFF (TNFSF13B) (57, 69). Of the TNFR family, silencing of *TNFRSF10A, TNFRSF10B*, and *TNFRSF1* did not alter *A. phagocytophilum* growth *in vitro*. However, *CD40, CD40L, TNFRSF11A* (RANK), *TNFSF14*, EDAR associated death domain (*EDARADD*), and baculoviral IAP repeat containing 3 (*BIRC3*) are significantly upregulated with *A. phagocytophilum* infection in several transcriptional profiling studies, and all are involved in the induction of or propagation of non-canonical NF-κB signaling (8, 14, 23). Non-canonical pathway-specific gene targets that are upregulated with *A. phagocytophilum* infection include both *CCL13*, which recruits inflammatory leukocytes except neutrophils, and *CCL19*, which impacts lymphocyte recruitment and homing in the thymus or lymph nodes.

Additionally, of the key TNFR signaling adaptors *TRAF2, TRAF3*, and *TRAF6*, expression is not significantly upregulated with *A. phagocytophilum* infection of HL-60 or human neutrophils (Supplementary Table 1C). NIK (MAP3K14) stabilization after TNFRs aggregate TRAF2, TRAF3 and/or TRAF6, the major adaptors for non-canonical signaling, permits their ubiquitylation by cIAP1/2 and degradation, ultimately allowing NIK to degrade IKKα (CHUK) and release of p100/p52 dimers in the non-canonical pathway. Here, silencing of both *TRAF3* and *TRAF6* leads to a more productive *A. phagocytophilum* infection. This finding further suggests that activation of non-canonical (or canonical) pathway is host-protective rather than an enhancer of bacterial fitness. While the finding does not inform about microbial fitness, it could be a potentially important observation given the recognition that non-canonical pathway activation is associated with unique transcriptional programs that in part guide lymphoid cell differentiation, dendritic cell function, and distinct inflammatory phenotypes, including chronic inflammatory conditions, autoimmune diseases such inflammatory bowel disease, acute kidney and lung injury, immunodeficiency syndromes, and even cancer (17, 69–72).

Of course, other possibilities exist for mechanisms by which *A. phagocytophilum* could influence NF-κB signaling. Ligation of innate immune receptors such as toll-like or NOD-like intracellular receptors can also lead to activation (73), and NLRC4 activates the inflammasome in *A. phagocytophilum* infection (74). We previously showed *A. phagocytophilum* triggered NF-κB-driven target gene expression through TLR2 but not TLR4 in primary *ex vivo* human monocytes (36). Prior transcriptional profiling of *A. phagocytophilum*-infected ATRA-differentiated HL-60 cells also demonstrated significant upregulation of *TLR2, TLR1, TLR3*, and *TLR6*, as well as *NOD2* and *NLRP3* (23). In fact, treatment of infected cells with wedelolactone and SC-514 led to increased transcription of *TLR1* and *TLR3*, but not *TLR2* in infected cells. The recent evaluation of differentially regulated NF-κB-related genes in response to various TLR ligands shows distinctive patterns for each TLR (75). When these genes were queried using the *A. phagocytophilum* transcriptional profiling in infected ATRA-differentiated HL-60 cells (23), the highest proportion of differentially expressed genes was found among the group observed when cells were stimulated by the TLR2 agonist, PAM-2CSK4 (data not shown). It is likely that responses triggered by PAMPs contribute to the sustained activation of NF-κB pro-inflammatory signaling in infected *A. phagocytophilum* cells. Finally, it is further recognized that cross-talk exists between the canonical and non-canonical pathways that could be regulatory or have other yet to be discerned purposes (72).

To synthesize a working model from these data, it is reasonable to start from the signal origin for ligands. The clear evidence of canonical pathway activation likely results from interactions with pathogen recognition receptors such as TLRs (36), NLRs (74), formyl-peptide and Fc receptors (40), or cytokines released from other stimulated cells that amplify the response. With regard to the non-canonical NF-κB pathway, signaling is initiated by ligands binding to TNFR superfamily members, for which there is evidence with *A. phagocytophilum* infection, including upregulated expression of several ligands and receptors. Although silencing of several of these TNFR superfamily members did not change *A. phagocytophilum* growth, with the possible exception of *LTBR*, of 8 TNFR superfamily genes detected in expression profiling of *A. phagocytophilum*-infected ATRA-differentiated HL-60 cells, 7 are upregulated from 2.9- to 178-fold, providing ample opportunity for non-canonical NF-κB signaling (23).

Aside from initiating signaling via PAMPs and related processes, pathogens can manipulate NF-κB signaling to their advantage, such as with viral RNAs that are detected by the cytoplasmic RNA sensor retinoic acid inducible gene I (RIG-I), or via production of viral proteins that interact with and alter various NF-κB signaling pathway proteins (18). Whether *A. phagocytophilum* or its effectors interfere with normal cellular functions or lead to transcriptional regulation of key NF-κB pathway genes is not proven, but given the evidence of activation of both canonical and non-canonical pathways, such extended studies might be fruitful. Despite increased *A. phagocytophilum* growth with silencing of some NF-κB signaling pathway genes, the ongoing differential transcription that yields significant upregulation of chemokine genes or those associated with suppression of apoptosis clearly benefit the pathogen, even if simply a component of physiologic NF-κB function. In fact, while the inhibitor and silencing data seem to argue for a protective role of NF-κB signaling, a majority of genes in these pathways are downregulated by *A. phagocytophilum* infection, reminiscent of the role that the type IV secretion system nuclear effector AnkA plays in dampening transcription of host defense genes, although no data here directly support that hypothesis (21, 41).

These observations provide evidence for NF-κB signaling via the canonical and non-canonical pathways as targets for control of infection or by which *A. phagocytophilum* could subvert host cell functions to impact microbial fitness. There are weaknesses in our study, including the lack of direct evidence of RELB in the nucleus of infected cells, and the ambiguity that is increasingly recognized in NF-κB signaling pathways that potentially confounded some studies leading to discrepant results. An increasing body of literature examines the cross-talk between the canonical and non-canonical NF-κB signaling pathways, and an emerging view is one of co-regulation and balance between acute and chronic inflammation and healing. Specifically how this occurs is still open for much additional research since inhibitors of NF-κB-mediated inflammatory suppression are in development (17). Moreover, an improved understanding of these inflammatory pathways could result from a clearer picture as to how *A. phagocytophilum* interacts with and reprograms its mammalian host cells while promoting a diversity of inflammatory responses that link closely to the diverse outcomes and human disease severity (4, 11, 76–78).

## Data Availability Statement

The datasets presented in this study can be found in online repositories. The names of the repository/repositories and accession number(s) can be found in the article/Supplementary Material.

## Author Contributions

JSD conceived and coordinated all research activities, interpreted results, and wrote the manuscript. ML conducted the majority of the experiments related to bacterial growth effects with pharmacologic inhibition and siRNA. W-HC conducted most of the experiments related to effects of pharmacologic inhibitors on transcriptional activity with infection. KR-B helped with the proteomics experiments and analyses. J-hP conducted and interpreted results from the initial experiments on NF-kappa B expression among nuclear and cytoplasmic preparations by protein immunoblotting. All authors contributed to the article and approved the submitted version.

## Conflict of Interest

The authors declare that the research was conducted in the absence of any commercial or financial relationships that could be construed as a potential conflict of interest.
