# Supplementary material for: Anaplasma phagocytophilum Activates NF-κB Signaling via Redundant Pathways
Source: Front Public Health. 2020 Oct 30;8:558283. doi: 10.3389/fpubh.2020.558283 (PMC7661751; doi:10.3389/fpubh.2020.558283)

Supplementary Material

## Supplementary Figures

**Supplementary Figure 1.** Drug toxicities for *A. phagocytophilum*-infected THP-1 cells or uninfected THP-1 cells. Mean normalized cytotoxicity was measured by comparing untreated to drug treated infected and uninfected cells separately Cytotoxicity greater than 2 or if significantly different on repeated studies were interpreted as toxic. NBD peptide was only tested for uninfected THP-1 cells and was not cytotoxic at 50 µM.


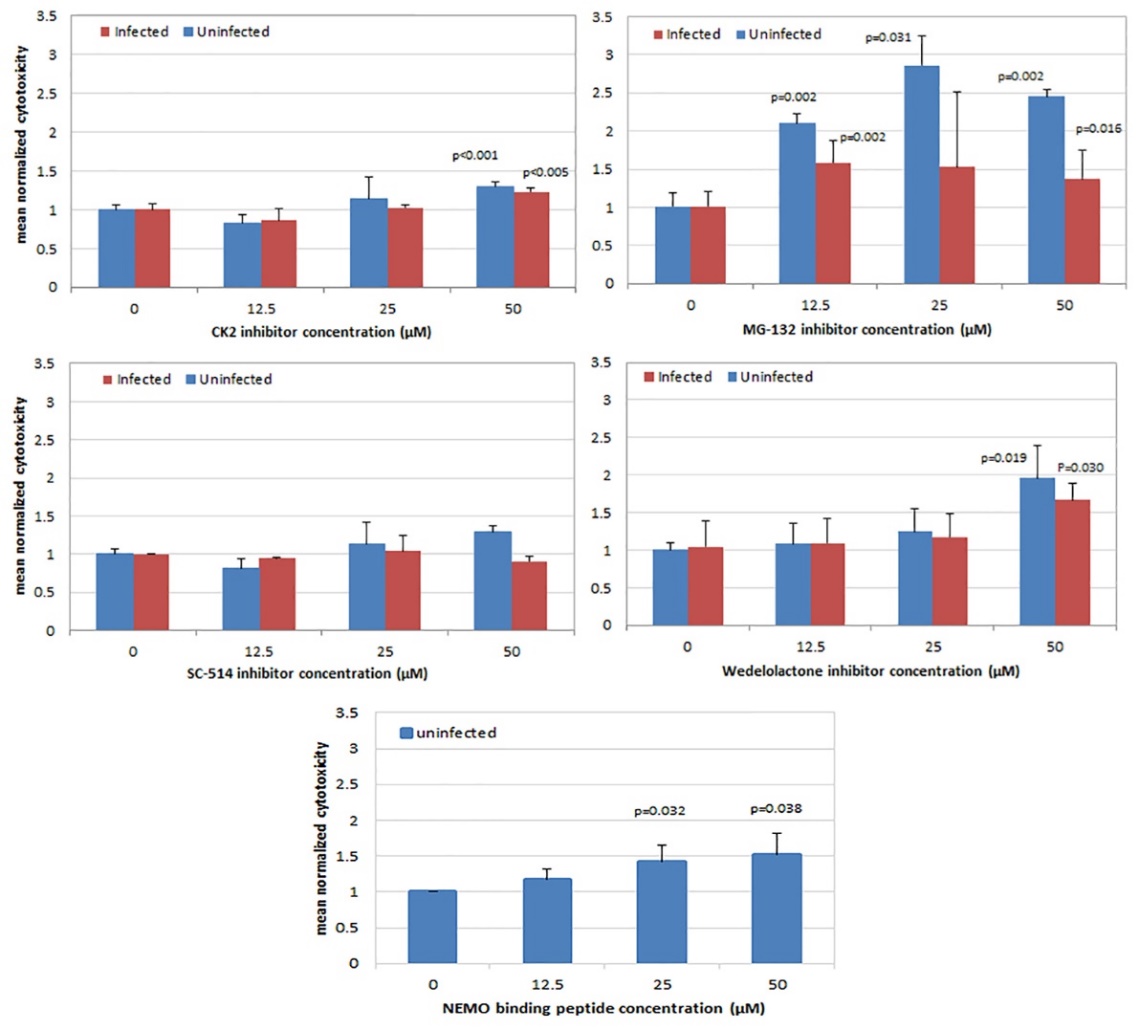


**Supplemental Figure 2**. Inhibition of non-canonical NF-κB activation with wedelolactone or SC-514 reverses suppression of many NF-κB signaling pathway genes transcriptionally dampened by *A. phagocytophilum* infection. Here, gene expression was normalized to that observed in uninfected THP-1 cells. Beyond the borders of the blue zone depicts >2 fold change compared to transcription in infected cells with or without the pharmacologic inhibitors. Note that a majority of those with differential transcription among this set of genes were downregulated by infection, and many of these were reversed by wedelolactone and SC514, pharmacologic inhibitors of the non-canonical pathway. Values that are significantly different (p<0.05) from uninfected cells are denoted by an asterisk (*).


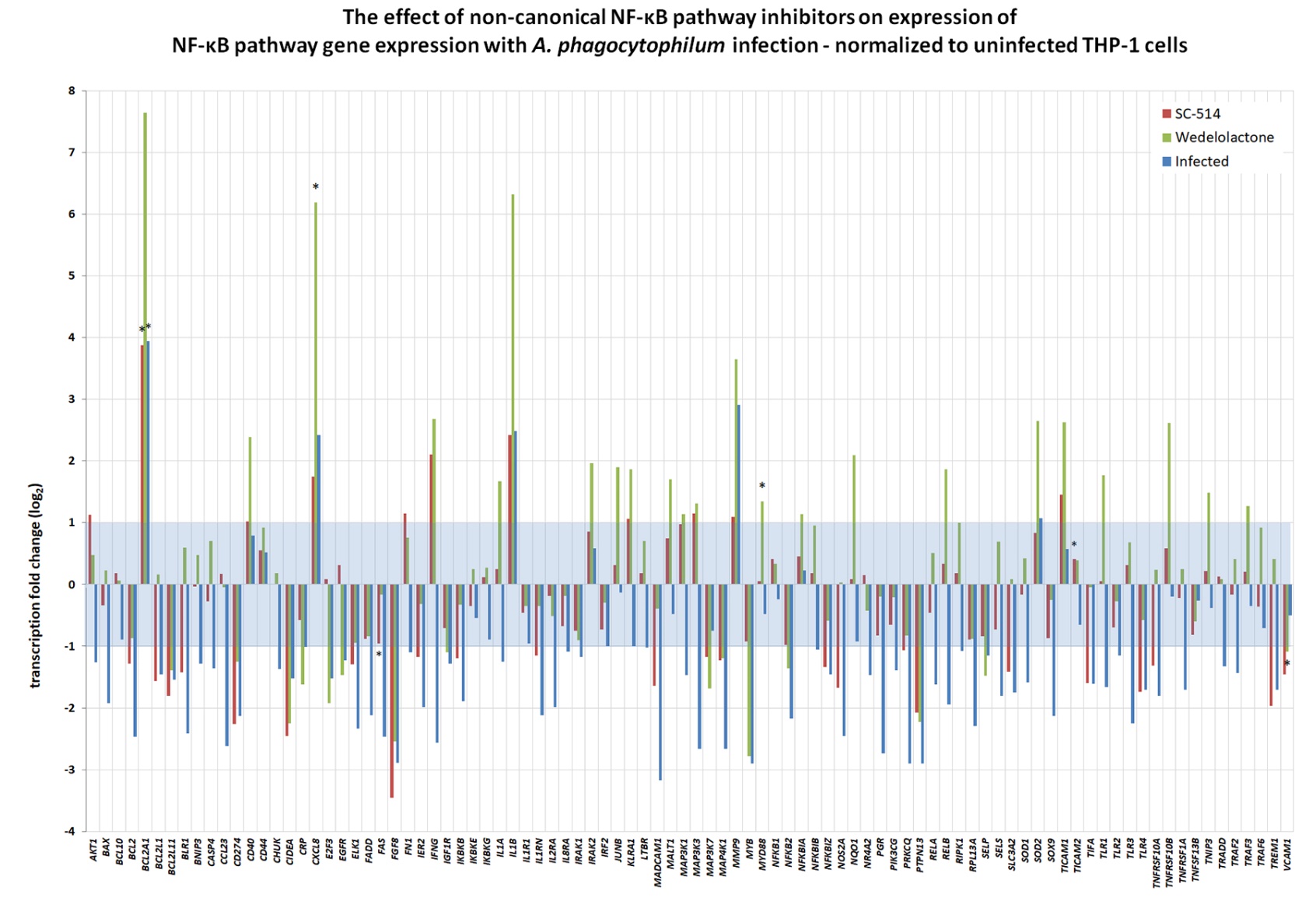

Supplement: Supplementary file 1 [file Data_Sheet_1.docx]
